# Human T-lymphotropic Virus Type 1 (HTLV-1)-Associated Uveitis Presenting With Frosted Retinal Vasculitis

**DOI:** 10.7759/cureus.115414

**Published:** 2026-08-29

**Authors:** Atsuki Fukushima

**Affiliations:** 1 Ophthalmology, Tsukazaki Hospital, Himeji, JPN

**Keywords:** autoimmune inflammation, corticosteroids, frosted retinal vasculitis, human t-lymphotropic virus type 1 (htlv-1), infectious disease, uveitis

## Abstract

Retinal vasculitis is a manifestation observed in various forms of uveitis. However, frosted retinal vasculitis, a severe type of retinal vasculitis, is seen in only a limited number of cases, typically associated with severe infections or autoimmune inflammatory conditions. A 65-year-old woman presented to our hospital after noticing a decline in vision in both eyes over the preceding month. Examination revealed vitritis and frosted retinal vasculitis in both eyes. Fluorescein angiography showed marked leakage from the optic disc and retinal vessels. Optical coherence tomography (OCT) revealed a serous retinal detachment at the right fovea. Sarcoidosis and Behçet's disease were ruled out based on blood tests, chest computed tomography (CT) scans, and the absence of extraocular symptoms or findings. There were no abnormalities in the white blood cell count or differential, and no elevation of C-reactive protein (CRP) was observed. While infections caused by various bacteria, viruses, and *Toxoplasma* were ruled out, elevated anti-human T-lymphotropic virus type 1 (HTLV-1) antibodies were detected. Based on these findings, the patient was diagnosed with HTLV-1-associated uveitis, and oral prednisolone therapy was initiated at a dosage of 30 mg. The vasculitis improved dramatically, and the dosage was gradually tapered while the condition remained stable. However, 16 months after the initial visit, the vasculitis worsened, necessitating an increase in the prednisolone dosage back to 30 mg. Subsequently, the dosage was tapered again, and treatment was discontinued 27 months after this resumption (43 months after the initial start of prednisolone). There was no recurrence of vasculitis thereafter, and the affected vascular areas transformed into degenerative lesions. Thus, HTLV-1-associated uveitis should be considered as a potential underlying cause of frosted retinal vasculitis.

## Introduction

In 1976, Ito et al. reported a pediatric case presenting with retinal vasculitis and named the condition "frosted branch angiitis" based on its characteristic findings in the Japanese language. Subsequently, similar cases were reported from Japan, many of which were of unknown etiology [1]. Although the pathogenesis remains unclear, frosted branch-like retinal vasculitis (frosted retinal vasculitis) has also been reported in cases of Eales' disease, a condition primarily characterized by retinal vasculitis [2].

Subsequently, there have been continued reports of frosted retinal vasculitis triggered by systemic infections. Early reports identified conditions such as tuberculosis [3] as potential triggers for frosted retinal vasculitis. More recently, infection-related conditions, including *Bartonella *infections [4], have been reported as triggers for frosted retinal vasculitis.

While many reported cases of frosted retinal vasculitis are associated with infectious diseases, there are also reports of onset secondary to non-infectious systemic inflammatory diseases. Initial reports involved conditions such as sarcoidosis [5] and multiple sclerosis [6]; more recently, it has been identified in patients with various autoimmune inflammatory diseases such as idiopathic juvenile rheumatoid arthritis [7]. Thus, it has become evident that both infectious diseases and autoimmune inflammation can trigger the onset of frosted retinal vasculitis.

Human T-lymphotropic virus type 1 (HTLV-1)-associated uveitis is a form of uveitis observed in HTLV-1 carriers; when first recognized as a distinct disease entity, it was referred to as "HTLV-1 uveitis" [8]. While the initial lesions in viral infections, such as herpes, are typically caused by the direct cytotoxicity of the virus itself, HTLV-1 is a retrovirus; consequently, apart from adult T-cell leukemia, the infection itself is unlikely to play a direct role in the pathogenesis of the associated conditions. Similar to other autoimmune-like inflammatory disorders associated with HTLV-1 infection, such as myelopathy [9], arthropathy [10], and thyroiditis [11], the condition is believed to stem from immune abnormalities rather than the viral infection itself, leading to its designation as "HTLV-1-associated uveitis" [12]. Subsequent animal studies have suggested that an autoimmune response may also play a role [13]; consequently, the condition has come to be recognized as an HTLV-1-associated disease, similar to myelopathy [9] and arthropathy [10]. Currently, a diagnosis of HTLV-1-associated uveitis is made if other known forms of uveitis are ruled out and the patient is confirmed to be an HTLV-1 carrier.

There have been no previous reports of frosted retinal vasculitis occurring as an underlying condition associated with HTLV-1-associated uveitis. We herein report a case of frosted retinal vasculitis observed in a patient with HTLV-1-associated uveitis.

## Case presentation

A 65-year-old woman became aware of decreased vision in both eyes one month prior and presented to our hospital. At the initial visit, best-corrected visual acuity was 0.9 (right) and 1.0 (left), and intraocular pressure was 22 mmHg (right) and 21 mmHg (left). Both eyes were pseudophakic (intraocular lenses implanted), and no signs of anterior segment inflammation were observed. However, anterior vitreous cells and bilateral retinal vasculitis with a "frosted branch" appearance were noted (Figure 1). 

**Figure 1:**
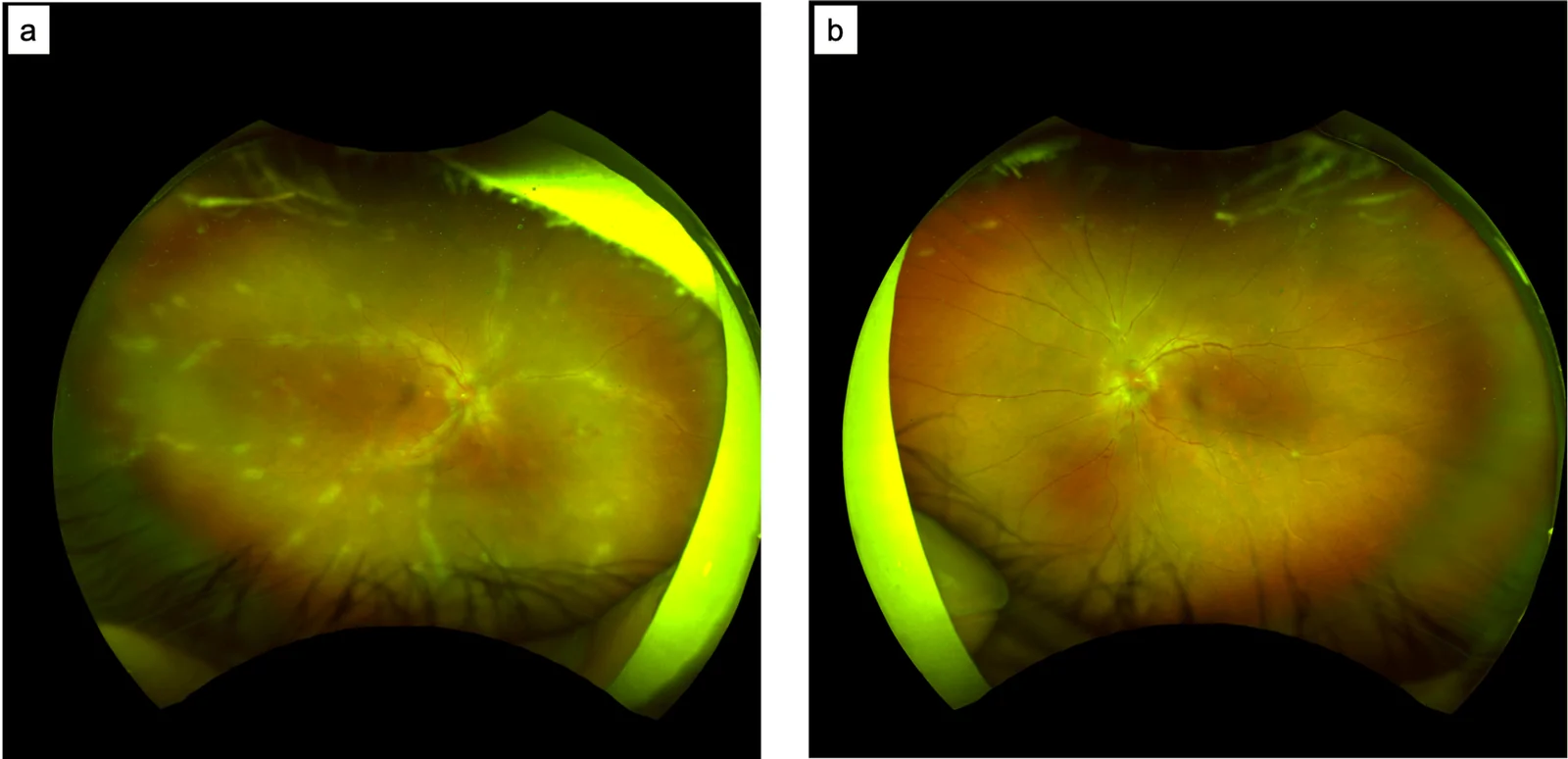
Fundus findings at the initial visit Color fundus photographs: (a) Marked exudates were observed along the retinal vessels of the right eye, presenting a "frosted branch" appearance. (b) Exudates were observed around the optic disc in the left eye.

Fluorescein angiography revealed marked leakage from the optic disc and retinal vessels (Figure 2).

**Figure 2:**
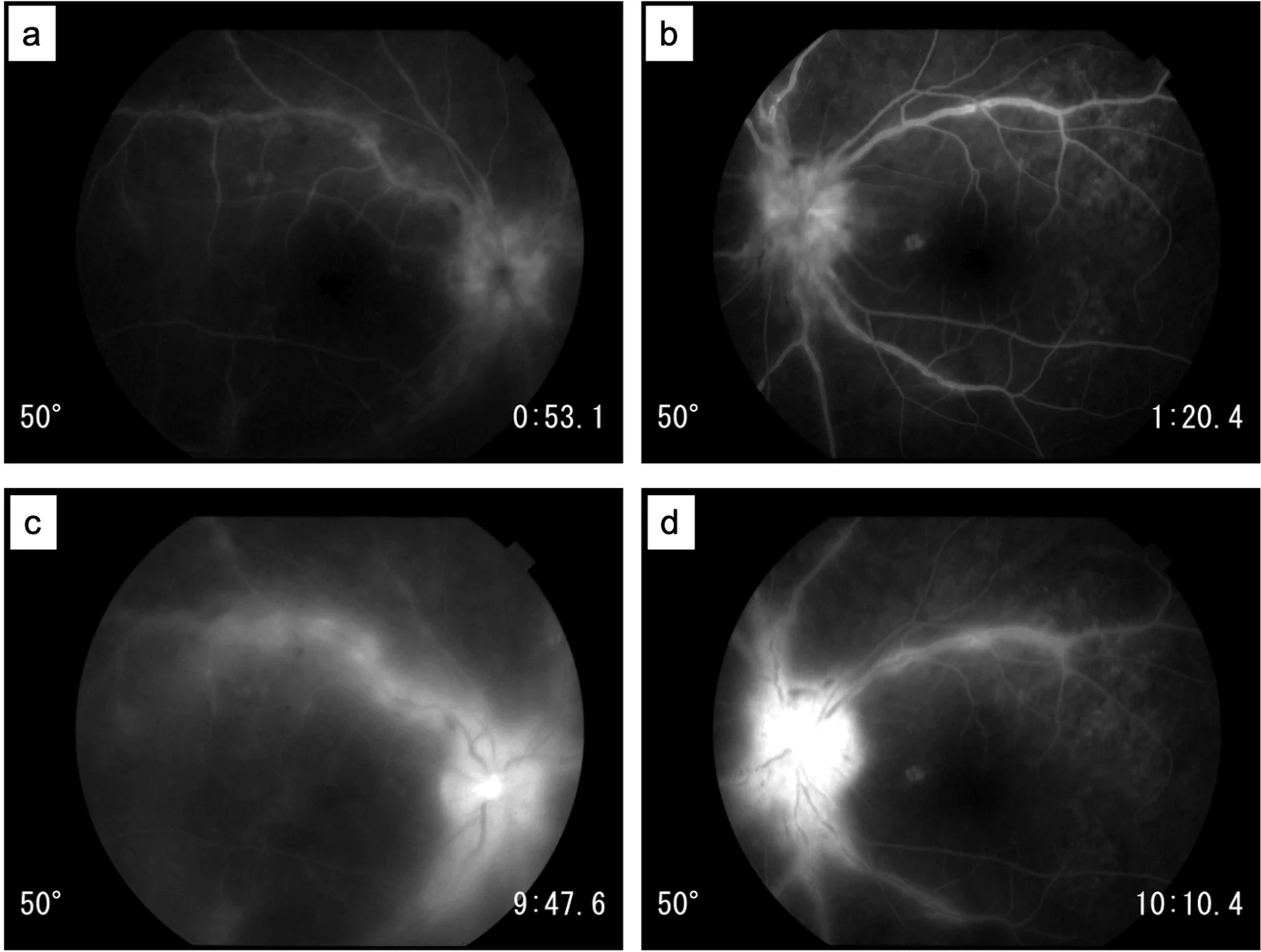
Fundus findings at the initial visit Fluorescein angiography images: (a, b) Images approximately 60 seconds after intravenous fluorescein injection. (c, d) Images approximately 10 minutes after injection. Leakage of fluorescent dye from the optic disc and retinal vessels was observed in both eyes, with intense staining noted at the 10-minute mark (c, d). Images of the right eye (a, c) and left eye (b, d).

Optical coherence tomography (OCT) showed a serous retinal detachment at the right fovea in the inferior region (Figure 3).

**Figure 3:**
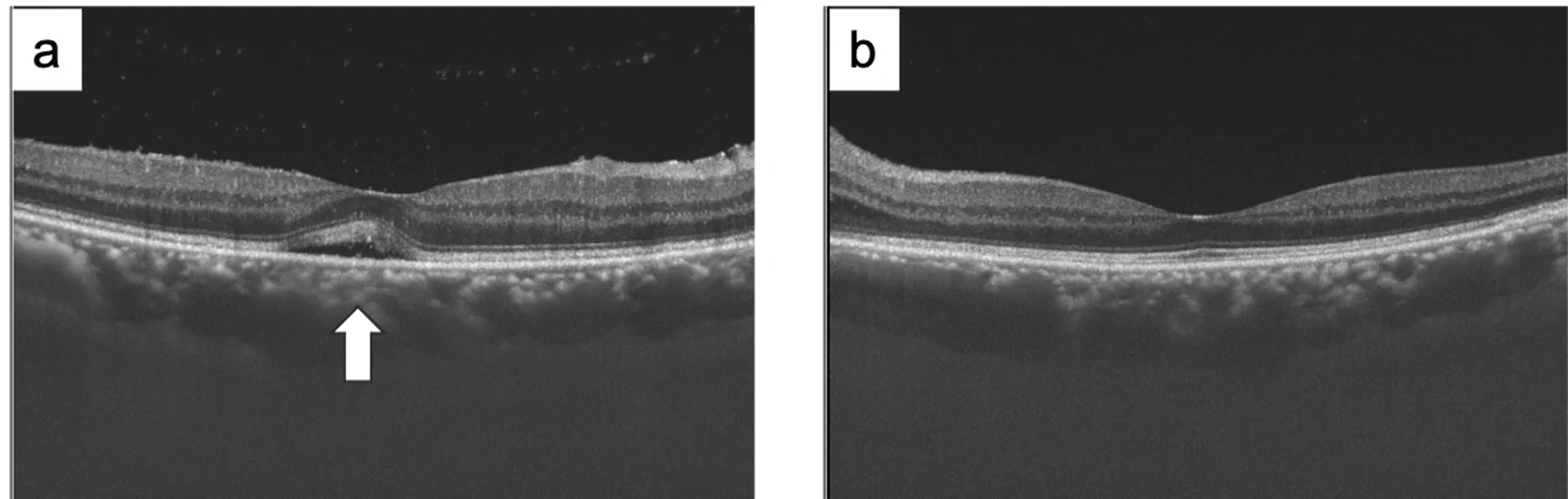
Fundus findings at the initial visit Optical coherence tomography (OCT) images: (a) Serous retinal detachment was observed at the fovea of the right eye (white arrow). (b) No abnormalities were observed in the left eye.

Sarcoidosis and Behçet's disease were ruled out based on blood tests, chest computed tomography (CT) scans, and the absence of extraocular symptoms or findings. Blood test results investigating the cause of uveitis, including white blood cell count and differential, serum calcium, C-reactive protein (CRP), angiotensin-converting enzyme, lysozyme, soluble IL-2 receptor, and IgM antibodies against herpes simplex virus, cytomegalovirus, varicella-zoster virus, and *Toxoplasma*, were all within normal limits. The only abnormal finding was elevated anti-HTLV-1 antibodies (2048-fold; normal range: 0-15-fold). Based on these results, the patient was diagnosed with HTLV-1-associated uveitis; oral prednisolone therapy was initiated at 30 mg, resulting in a dramatic improvement of the vasculitis (Figure 4).

**Figure 4:**
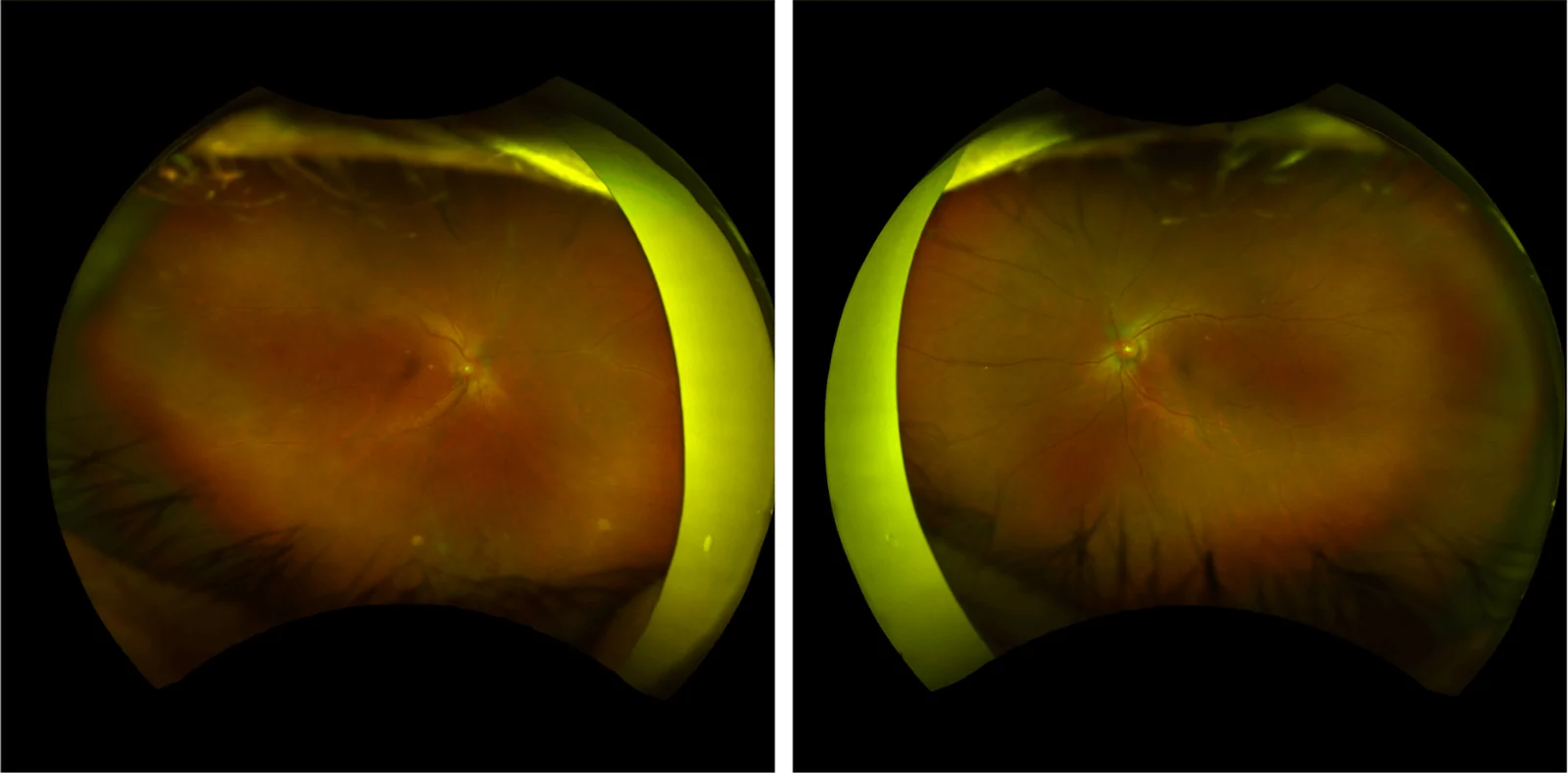
Color fundus photograph five weeks after the start of treatment Improvement of retinal vasculitis was observed in both eyes.

The dosage was gradually tapered; by 15 months after the initial visit, the patient was stable on a reduced dose of 5 mg of prednisolone. However, in the 16th month, a worsening of the vasculitis was observed (Figure 5), and the prednisolone dosage was increased back to 30 mg, although OCT imaging showed no signs of serous retinal detachment or intraretinal fluid. 

**Figure 5:**
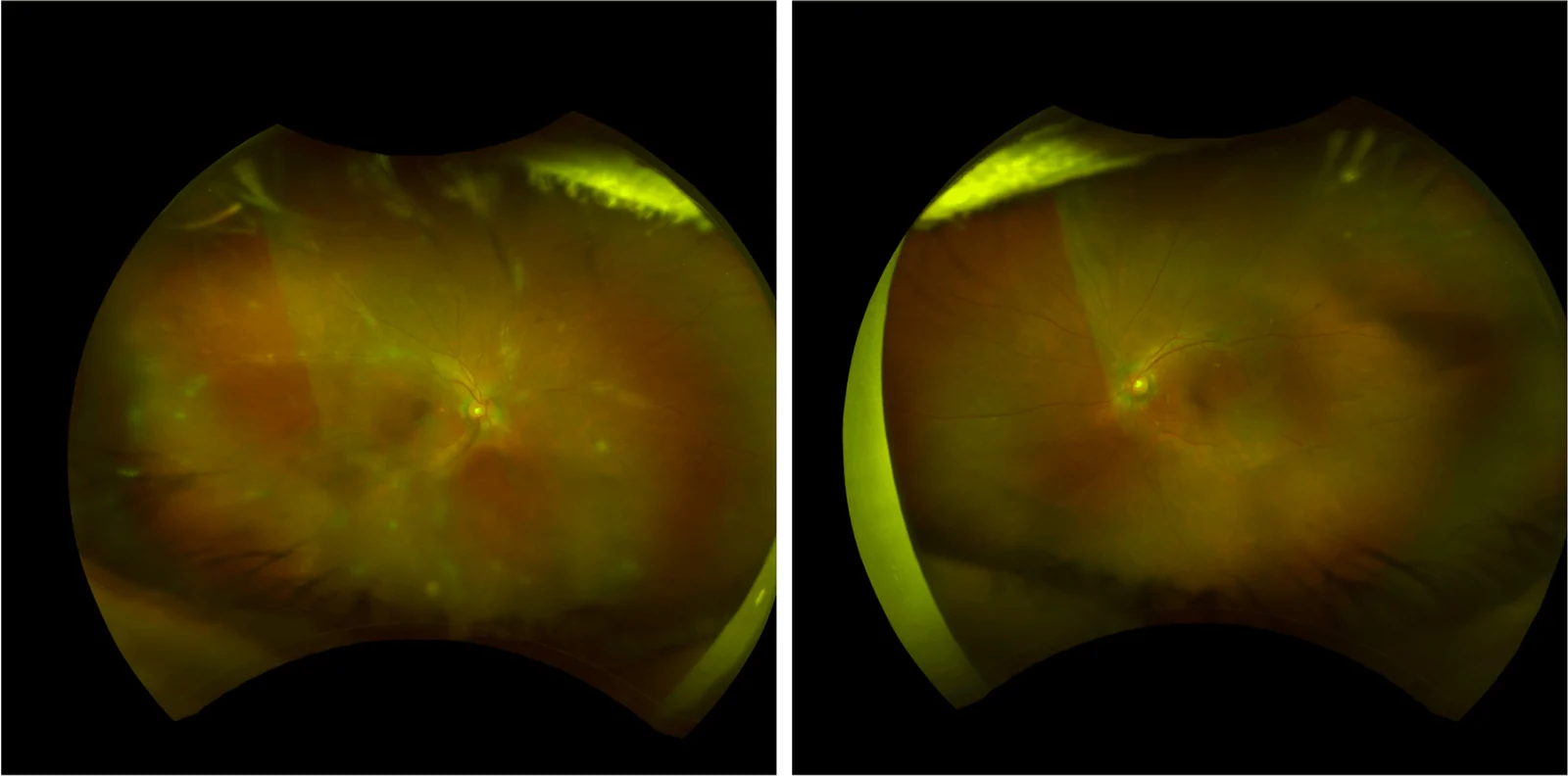
Color fundus photograph 16 months after the start of treatment Recurrence of retinal vasculitis was observed in the right eye.

Subsequently, the prednisolone was tapered again, and treatment was discontinued 27 months after this resumption (43 months after the initial start of prednisolone). There was no recurrence of vasculitis thereafter; at the final visit seven years after the initial consultation, corrected visual acuity was 1.5 in both eyes, and intraocular pressure was 19 mmHg (right) and 23 mmHg (left), showing no deterioration, while the site of the vasculitis had transformed into a degenerative lesion (Figure 6).

**Figure 6:**
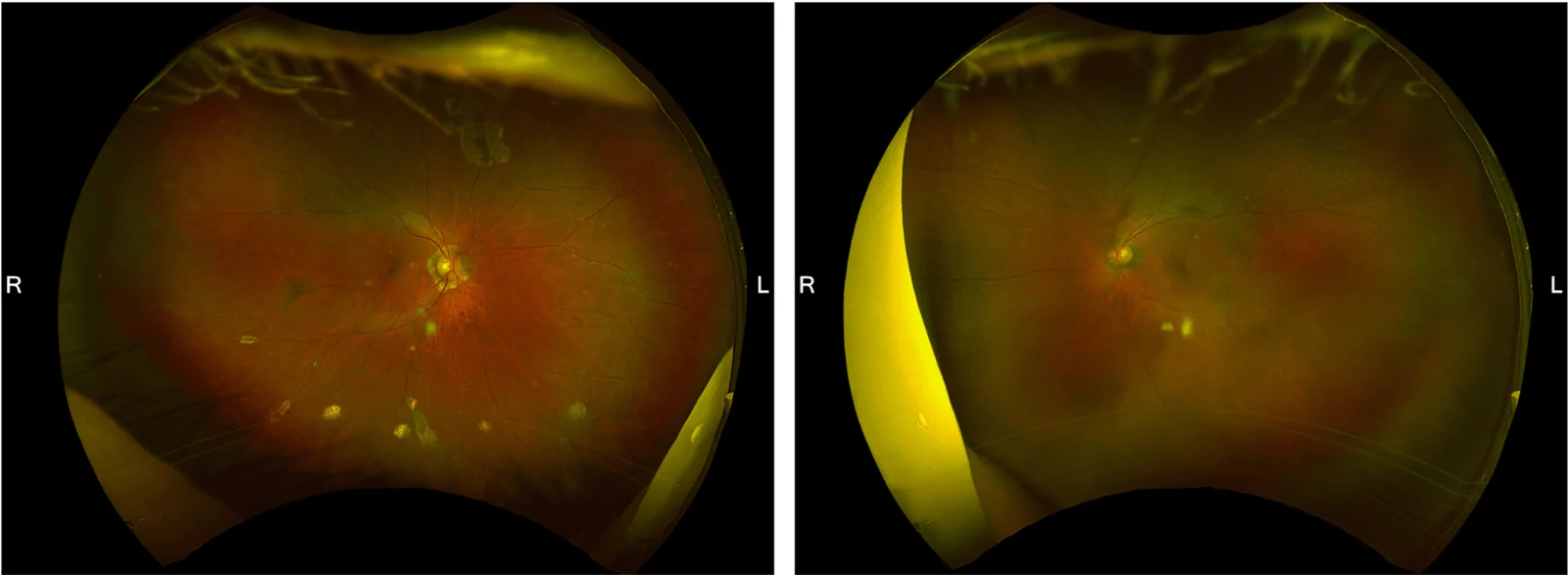
Color fundus photograph seven years after the start of treatment (41 months after the discontinuation of oral prednisolone) Retinal vasculitis had resolved in both eyes, and areas of perivascular degeneration were observed.

## Discussion

HTLV-1-associated uveitis has been defined as a form of uveitis that is seropositive for HTLV-1 but lacks an identifiable underlying cause, essentially an unclassifiable type of uveitis, and its clinical characteristics have been analyzed [12]. Reported characteristics include the following: (1) onset as acute uveitis, commonly presenting with blurred vision and floaters, in middle-aged adults, with no gender predilection; (2) frequent association with vitreous opacity and retinal vasculitis, though exudative changes in the retina and choroid are rare; (3) a favorable response to corticosteroids; (4) onset in one or both eyes, with a high rate of recurrence within a few years; (5) a generally good visual prognosis; (6) potential coexistence with HTLV-1-associated myelopathy or hyperthyroidism; and (7) serum HTLV-1 antibody titers that are significantly higher than those found in HTLV-1 carriers. Our case met all these criteria except for item 6. Although there were some minor differences in characteristics, the clinical findings were generally consistent with the aforementioned report [12], even in regions with low HTLV-1 prevalence [14].

Although retinal vasculitis is observed in HTLV-1-associated uveitis, its severity is generally considered mild [12]. A report from France noted that among 175 patients diagnosed with adult T-cell leukemia/lymphoma, three exhibited intraocular findings associated with the disease [15]. All three cases presented with bilateral deep retinal infiltration accompanied by intermediate uveitis and severe retinal vasculitis; the findings suggested that infiltration by leukemic cells was the underlying cause. There has also been a report of frosted retinal vasculitis in a patient with adult T-cell leukemia [16]. In that instance, the condition was attributed not to direct infiltration by leukemic cells, but rather to an immune response triggered by allogeneic hematopoietic stem cell transplantation. It is highly probable that the frosted retinal vasculitis in our case developed through a similar mechanism.

In addition to uveitis, a report from Brazil has documented cases of keratoconjunctivitis sicca associated with HTLV-1 [17]. Recent studies indicate that approximately one-third of HTLV-1-infected individuals develop keratoconjunctivitis sicca, with a particularly high risk observed in patients with HTLV-1-associated myelopathy [18]. Thus, HTLV-1 infection can affect tissues beyond the uvea, potentially leading to conditions such as inflammatory keratoconjunctival lesions. This evidence suggests that HTLV-1 has the potential to trigger autoimmune-mediated inflammation, in addition to its role as an infectious agent.

## Conclusions

While retinal vasculitis is a manifestation of uveitis, frosted retinal vasculitis, a severe form of retinal vasculitis, is observed in only a limited number of cases, typically associated with severe infections or autoimmune inflammation. We encountered a case of frosted retinal vasculitis in a patient with HTLV-1-associated uveitis. When encountering uveitis accompanied by retinal vasculitis, HTLV-1-associated uveitis should be included in the differential diagnosis. If frosted retinal vasculitis is observed, it is necessary to monitor the patient while keeping in mind the potential onset of HTLV-1-associated uveitis as well as adult T-cell leukemia/lymphoma.
